# Trauma Education and Stigma Reduction in Global Settings: An Evaluation of the Impact of a One-Day Trauma Psychoeducation Workshop with Community Stakeholders in the Caribbean Nation of Saint Lucia

**DOI:** 10.3390/ijerph17072255

**Published:** 2020-03-27

**Authors:** Anu Asnaani, Su-Anne R. Charlery White, Ifrah Majeed, Tammi-Marie Phillip

**Affiliations:** 1 University of Utah, Salt Lake City, UT 84112, USA; 2 HERStoire Collective, Castries LC01, Saint Lucia; 3 Butler Hospital, Providence, RI 02906, USA

**Keywords:** trauma, psychoeducation, stigma reduction, low resource/global settings

## Abstract

The independent island nation of Saint Lucia and surrounding Caribbean countries have fairly well-documented high reported rates of trauma, but limited training infrastructure for trauma-related mental health support and treatment services. This study addresses this disparity between high trauma exposure and sparse trauma-related resources by studying how a one-day training workshop impacted self-rated knowledge about trauma and stigma towards trauma survivors. The training was provided by a licensed clinical psychologist in partnership with a local women’s rights group. Participants (*n* = 41) included school counselors, nurses, psychiatric providers, health educators, and advocates on the island. Participants completed pre- and post-workshop measures examining the variables of interest. The one-day workshop provided training on trauma types, post-trauma reactions, options for treatment, and hands-on training for trauma crisis-management and short-term interventions. Following the workshop, participants reported increased knowledge of trauma, more accurate perceptions of its prevalence, better understanding of evidence-based treatments, and lower trauma survivor-related stigma. This is the first trauma-focused workshop tested in St. Lucia, where the need for such training is considerable given few treatment options for trauma survivors in this area. Work is underway to provide more expansive services for trauma across the Caribbean region, given these preliminary promising findings.

## 1. Introduction

It is estimated that nearly 70% of the population across the globe will experience at least one traumatic event (e.g., physical/sexual abuse, natural disasters, automobile/man-made accidents, gun or other violence) in their lifetimes [1], and approximately 10–15% of individuals experiencing such distressing events will go on to develop significant mental health problems as a result, such as post-traumatic stress disorder (PTSD), generalized anxiety disorder (GAD), specific phobias, depression, substance use disorder, and other related mental health issues [2,3]. In addition, the occurrence of these mental health issues following exposure to a traumatic event is associated with significant healthcare burden and distress for those experiencing such events, including greater number of days missed from work, significantly higher overall healthcare costs, and a distinct reduction in quality of life and life functioning [4,5,6]. In particular, trauma exposure is notable in the Caribbean region, which consists of some 13 sovereign countries (in addition to a dozen other territories) and some 45 million individuals across the entire region, with studies finding elevated prevalence rates and subsequently greater functional impairment due to common mental health consequences of trauma exposure (such as PTSD, suicidality, and drug use, particularly in women and adolescent survivors of trauma) specifically for individuals coming from this island archipelago [7,8,9].

In contrast, the current rates of access to effective and evidence-based treatment options for trauma survivors in the Caribbean remain at unknown levels, both in urban and rural parts of these countries. The World Health Organization (WHO) conducted an evaluation of mental health systems, providers, and legislation across the wider Latin American and Caribbean region, and noted a very low number of psychologists or psychiatrists in proportion to the populations in the individual countries in the region [10]. Further, there is little to no published data on current *evidence-based* mental health practice utilization or implementation rates in the region, which is partially due to significantly lacking resources around conducting quality research to examine such a phenomenon in the majority of countries in the region [11,12]. The problems around access to adequate resources for trauma survivors are multi-fold, and closely mirror the ongoing issues related to health disparities observable specifically for Caribbean/other immigrant populations even within North America and Europe [13,14], where treatment options are otherwise generally better overall than in the Caribbean. For instance, there is a major issue related to having an adequate number of trained providers to offer evidence-based treatments for post-trauma psychological sequelae in the Caribbean and for Caribbean immigrant groups living abroad [15], which is a major barrier to meeting the needs of trauma survivors. Furthermore, the majority of the limited providers who do exist in these settings have not been given the opportunity to be trained in effective treatments themselves, and the few who have had such training reside primarily in the capital cities or only in a few of the largest nations in this region [15,16] which greatly reduces access for rural or village-dwelling survivors of trauma, further strengthening the existing health disparities in this domain.

As a way to meet this growing divide between Caribbean survivors of trauma and provision of evidence-based care for these individuals, it is paramount to find ways to (1) increase the number of providers trained in such effective treatments [17], and (2) to engage in the task-shifting efforts seen in other global settings to expand the range of frontline providers and stakeholders who can support trauma survivors from only mental health counselors or therapists to non-mental health specialists such as school counselors, advocates, public health educators, and medical providers in primary care [18]. Indeed, multiple efforts by research scientists in other global contexts have pointed to the utility of expanding training efforts to such a broader array of potential providers in order to significantly reduce disparities related to access and outcome following trauma exposure in lower resource or non-Western settings [19,20]. In addition, relevant to the current study, there has been no documented study to date that has specifically examined the effectiveness of a training program to such a range of front-line providers in the Caribbean, highlighting the continued need for such work in this region.

It is also important to consider that task-shifting efforts are not without their challenges; several previous attempts in this domain in other global settings have faced obstacles related to reluctance to implement evidence-based treatments and lack of systemic or leadership support for training of a wider range of providers in evidence-based mental health treatments [17]. Indeed, given the lack of systematic evaluation of such training efforts in evidence-based psychological therapies for trauma survivors in the Caribbean, there are considerable gaps in understanding the current state of provider and stakeholder knowledge about trauma and PTSD (the most prevalent psychological disorder stemming from trauma exposure [21]), familiarity with existing evidence-based treatments for survivors of trauma, and ongoing stigma or biased/blaming beliefs about trauma survivors. In the Caribbean specifically, there remains ongoing stigma about mental health and trauma more broadly in the general population ([13], see [22] for a review), with a low base rate (less than 50%) of individuals with an identified mental health problem seeking treatment for any mental health problem overall [23] and inequitable access to mental health resources and treatments across the region [24], which further compounds the problem of adverse outcomes for trauma survivors specifically in these countries. Such ongoing stigma and low treatment-seeking rates from mental health specialists in this region further underscores the importance of training a wider range of stakeholders whom trauma survivors may be more willing to approach, and training these individuals to have more accurate beliefs and knowledge around the types of problems survivors might seek their assistance on.

This study, therefore, aimed to bridge some of these gaps in the literature by examining the effectiveness of a one-day training workshop in improving knowledge around trauma, its prevalence, and effective psychological treatments, while reducing stigma towards trauma survivors in a wide range of stakeholders engaged in treatment and support of trauma survivors in Saint Lucia. St. Lucia is an independent island nation in the Caribbean, with a geographical setup, demographic makeup, and education system very similar to a number of surrounding countries in the region. This workshop was designed to be the first in what is becoming an annual training effort on the island and in the surrounding region, and was intended to provide an initial sense of need and interest in trauma training in the area. Quantitative data and qualitative feedback obtained before and after the workshop were analyzed in the current study to provide currently lacking information about knowledge and stigma about trauma, within the context of a brief training workshop.

## 2. Methods

### 2.1. Participants

Participants (*n* = 41) were composed predominantly of adult women (*n* = 34) and were predominantly college educated (*n* = 31). Most participants were in their late 30s (*n* = 32) and had on average between 3–10 years of prior experience working with trauma survivors in various capacities (see Table 1). The most frequent occupations reported by participants were school guidance counselor (*n* = 12), non-governmental organization (NGO) representative/advocate (*n* = 10), and registered medical nurse (*n* = 8).

Participants were recruited for the study using flyers distributed to medical providers, psychiatric providers, school counselors, and other governmental bodies via the Ministry of Health and Wellness in St. Lucia. Flyers were also distributed to specific advocacy groups identified as appropriate for the trauma training, who were allowed to pass on this flyer to other interested providers. Participants were provided with a contact email to indicate their interest in attending this training workshop. The workshop was conducted in close collaboration with the HERStoire Collective, a sexual and reproductive health advocacy group based in the Caribbean, which provided support for the workshop in terms of facilitation with other governmental bodies and NGOs on the island, provided guidance around study procedures, weighed in on workshop content, provided physical assistance on the day of the workshop, and assisted in the media coverage of this event.

While approximately 100 individuals indicated interest in obtaining this training, only 43 could be accepted into the workshop due to limited space and funding allocations, while the rest were informed of their placement on a waitlist for any future trainings that may be conducted in this area. The final sample includes those workshop attendees (*n* = 41) who verbally consented to providing their deidentified data for the purposes of this study. Study procedures were reviewed and approved by the University of Utah Institutional Review Board, which qualified this study as a quality assurance/quality improvement project given its focus on determining the utility of a one-day training workshop for the target group of attendees in this setting.

### 2.2. Measures

**Figure 1:**
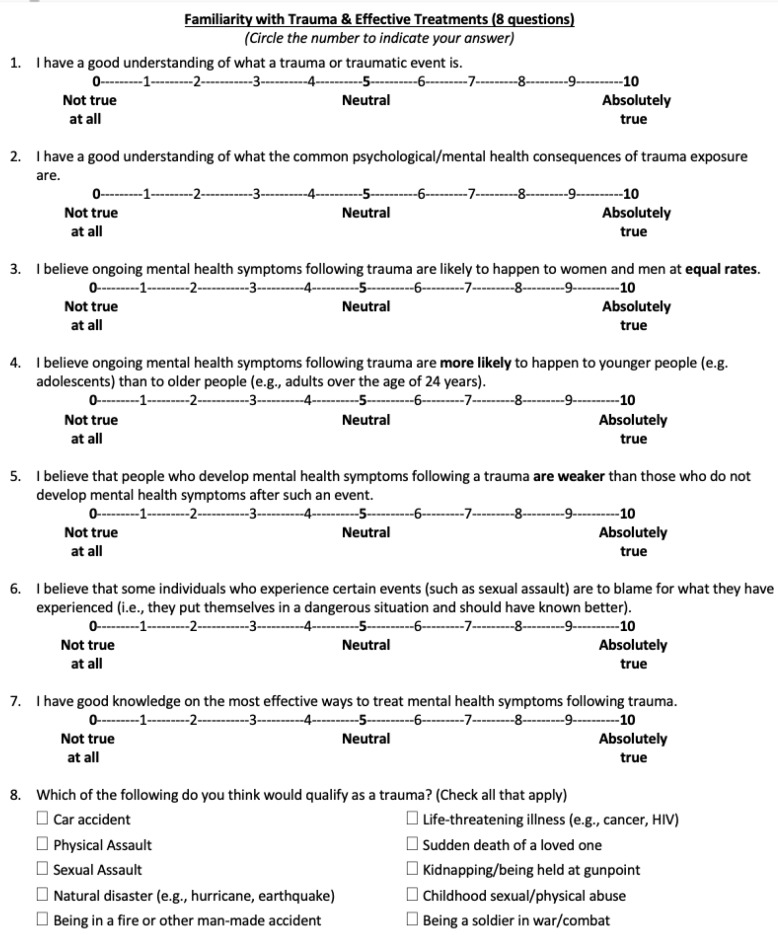
Knowledge/stigma about trauma and effective treatments questionnaire items.

*Knowledge about trauma and effective treatments*. This was an eight-item self-report questionnaire composed by the author team specifically for the purposes of this workshop. The survey was divided into four subscales that indicated self-reported (1) knowledge about trauma (items 1–2 and 8), (2) beliefs regarding trauma prevalence (items 3–4), (3) familiarity with effective trauma treatments (item 7), and (4) stigma towards trauma survivors (items 5–6). Each question was rated using a 10-point Likert scale, with higher scores indicating greater knowledge about trauma, its prevalence, and effective treatments, and lower stigma towards survivors. This questionnaire was completed by participants before the start of the workshop and again at the end of the workshop. See Figure 1 for specific question prompts.

**Figure 2:**
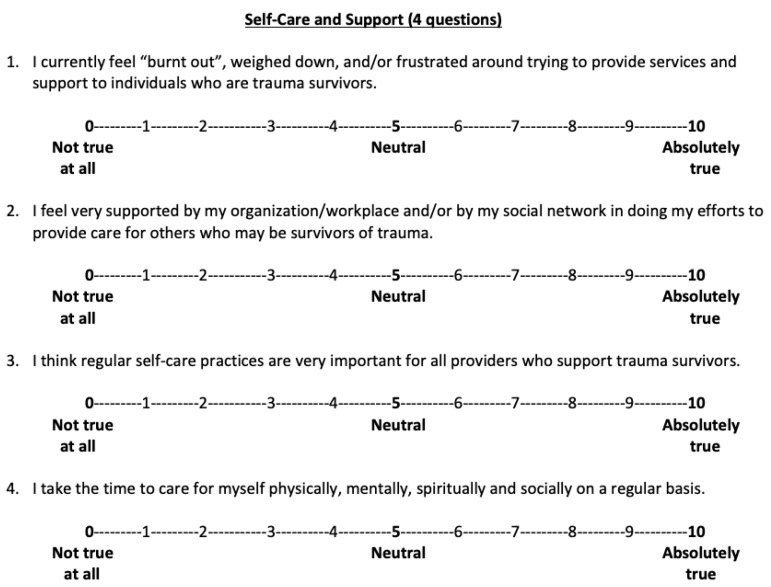
Self-care and support questionnaire items.

*Self-care and support*. This was a four-item self-report questionnaire that was also created specifically for this workshop by the author team. This self-report was used to measure participants’ attitudes regarding self-care prior to the start of the workshop only. Each question was rated using a 10-point Likert scale, with higher scores indicating better self-care attitudes and behaviors, with the exception of question 1 where higher scores indicated greater feelings of burnout. See Figure 2 for specific question prompts.

*Feedback about workshop.* Numerically rated questions and open-ended, qualitative data were also collected at the end of the workshop from each participant to evaluate the usefulness of the workshop, and also, in part, to understand the community’s interest and other potential areas of need that may not have been covered by the current workshop. Feedback was collected using two questions using a 10-point Likert scale assessing for the success of the workshop in meeting its goals (to increase knowledge about trauma and its effective treatments, and effective ways to engage in self-care), and three open-ended questions with written feedback specifically on (1) what aspects participants found helpful about the workshop, (2) what could be improved, and (3) additional topics and training that would be useful for participants to receive further training on in the future.

### 2.3. Procedure

Prior to the start of the workshop, participants were asked to provide their demographic information and complete other pre-workshop measures (assessment of knowledge on trauma, prevalence, treatments and stigma, and self-care beliefs/practices).

The one-day workshop followed a detailed hourly agenda, and provided attendees with psychoeducation on types of trauma, typical post-trauma reactions, and detailed information on one of the front-line evidence-based PTSD treatments (prolonged exposure, PE [25]) while also providing hands-on training for handling immediate trauma crisis, specifically, psychoeducation on common reactions and breathing retraining, both taken from the PE manual [25], and a variety of distress tolerance techniques such as specific dialectical behavior therapy skills [26] and progressive muscle relaxation [27]. The workshop ended with a session on self-care practices (including education on mindfulness as a skill with practice in the session [28]) and small group break-out discussions on ways to engage in this practice more regularly to prevent burnout. The training was provided by the first author (A.A.) in close partnership with HERStoire and the second author (S.R.C.W.) around the most helpful content to provide, given the heterogeneity of stakeholders attending. In addition, the fourth author (T.M.P.), who is a trained psychiatrist, joined the training via Zoom to provide additional feedback and guidance on the topics discussed during one of the morning sessions.

Prior to the collection of the post-workshop questionnaires, all attendees were informed of the intent to use the deidentified data from the questionnaires for assessment of effectiveness/usefulness of the workshop and were given the option to not provide their completed questionnaires. Two attendees did not wish to participate in the study and did not provide this post-workshop data. Their pre-workshop data was, therefore, omitted from the dataset, while the remaining 41 participants completed the post-workshop questionnaires and voluntarily turned in this data to indicate consent to participate. Participants were not compensated for their completed questionnaires; however, the workshop and food for the day were provided to all attendees (regardless of questionnaire completion) free of charge.

### 2.4. Data Analysis

This study used descriptive, correlational, and regression analyses, in addition to qualitative data analysis of the workshop feedback. Descriptive analyses (mean, standard deviation (SD) or frequencies) were used to analyze the participants’ demographic data and baseline levels of trauma knowledge/stigma and self-care as reported before the workshop. Correlational analyses were conducted among the subscales of the questionnaire examining trauma knowledge and stigma and demographic variables before the workshop, in order to identify any variables that would need to be accounted for in subsequent regression analyses. For regression analyses, a one-way repeated measures analysis of variance (ANOVA) was used to examine how participant knowledge of trauma, trauma treatment, trauma prevalence, and stigma towards trauma changed before and after attending the trauma workshop. Any potentially confounding demographic or baseline variables discovered in the correlational analyses were controlled for as covariates in the first step of the ANOVA analysis, followed by the predictor of interest in the second/final step of the model. Each subscale was analyzed separately, and subscales that did not reveal any significant relationships in the correlational analysis were not controlled for.

Qualitative and quantitative data regarding the usefulness of the workshop were analyzed at the end of the workshop. First, descriptive analyses (mean, SD) were conducted on the two Likert-scale items pertaining to usefulness of the workshop content in increasing knowledge about core concepts. This was followed by thematic analysis with numerical coding techniques. This coding was completed by two trained research assistants who had to come to a consensus on each participant’s open-ended qualitative data about the major themes related to participants’ perspectives on the positive aspects of the workshop, areas in need of improvement, and future areas in need of further training in this group of stakeholders. This coded data and the resulting themes identified for each response were then checked for consistency and accuracy by the first author (A.A.) and third author (I.M.).

## 3. Results

### 3.1. Descriptive Analysis

Most participants reported self-care practices as being very important for individuals supporting trauma survivors (Mean (M) = 9.46/10, SD = 1.21) and many reported engaging in some physical, mental, spiritual, or social self-care on a regular basis (M = 7.15/10, SD = 1.83). Prior to engaging in the intervention, participants felt fairly knowledgeable regarding trauma definitions (M = 24.00/30, SD = 4.49), somewhat knowledgeable about trauma prevalence (M = 10.67/20, SD = 3.38), and somewhat knowledgeable about evidence-based trauma treatments (M = 5.13/10, SD = 2.26), and attached fairly low stigma to trauma (M = 3.85, SD = 4.23).

### 3.2. Correlational Analysis

Correlational analyses did not reveal age or education level to be significantly correlated with any of the trauma subscales (see Table 2). However, prior experience with trauma survivors was significantly (r = 0.531, *p* < 0.01) correlated with participants’ self-reported knowledge about trauma, such that participants with greater prior experience with trauma survivors reported being more knowledgeable about trauma. Prior experience with trauma was, therefore, controlled for in our regression analysis examining this knowledge of trauma subscale. In terms of other significant associations, age was unsurprisingly found to be significantly (r = 0.343, *p* < 0.05) correlated with higher education level, such that older participants were more likely to have a higher education level. Participants with higher education levels were also significantly (r = 0.395, *p* < 0.05) more likely to have greater experience working with trauma survivors. Finally, knowledge about trauma was also significantly correlated with placing a higher value on self-care practices (r = 0.683, *p* < 0.001), and greater engagement in regular self-care behaviors (r = 0.385, *p* < 0.05).

### 3.3. Regression Analysis

A one-way repeated measures ANOVA analysis was used to measure differences in participant self-rated trauma knowledge and stigma subscales before and after the workshop (see Table 3). Even after controlling for prior experience with trauma, participants reported significantly increased knowledge about trauma (Wilks’ Lambda = 0.83, *F* (1, 24) = 5.11, *p* < 0.05, effect size (ES) = 0.18) after the workshop. Similarly, participants also reported significantly more accurate perceptions of trauma prevalence (Wilks’ Lambda = 0.70, *F* (1, 34) = 14.64, *p* < 0.005, ES = 0.30), a lower stigma towards trauma survivors (Wilks’ Lambda = 0.85, *F* (1, 37) = 6.58, *p* < 0.05, ES = 0.15), and more self-rated knowledge regarding effective trauma treatments (Wilks’ Lambda = 0.32, *F* (1, 35) = 74.54, *p* < 0.0005, ES = 0.68) after the workshop.

### 3.4. Feedback about Workshop

**Figure 3:**
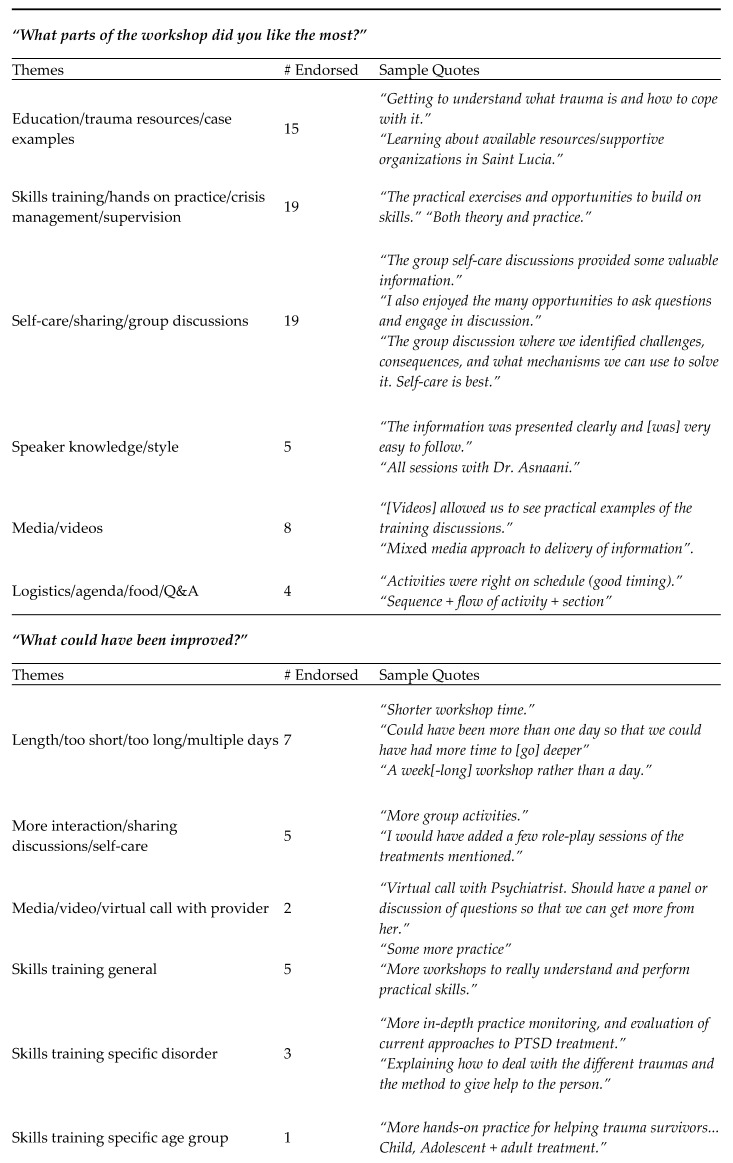

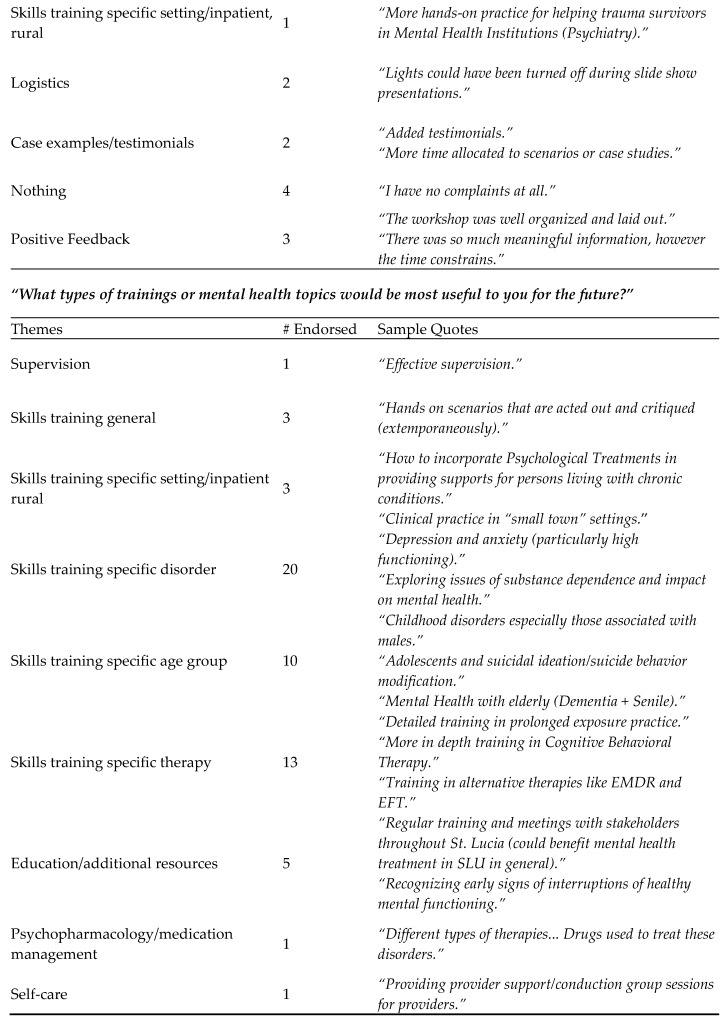
Major themes derived from analysis of qualitative workshop feedback. Note: Minor typos and spelling errors were corrected.

Participants generally reported very high average ratings pertaining to self-rated increase in knowledge about trauma and its effective treatments (M = 9.10/10, SD = 1.00) and ways to engage in self-care (M = 9.10/10, SD = 0.94). Thematic analysis of the qualitative data revealed interesting major and frequently occurring themes for each of the three open-ended questions (see Figure 3). For instance, the most frequently endorsed positive aspects of the workshop were identified as the skills-training and hands-on nature of the workshop (*n* = 19), and the self-care/group discussions around this self-care (*n* = 19). Participants were more mixed with their feedback on what could have been improved about the workshop (with only about half of respondents providing any critical feedback for this question at all), but the most frequent themes emerged around the workshop not being long enough as a one-day only workshop (*n* = 7), the need for more interactive/discussion-based activities (*n* = 5), and the need for training in more general mental health treatment skills (*n* = 5). In terms of future topics, about half of the participants requested training for specific disorders (*n* = 20) such as depression, anxiety disorders, and substance abuse, or training on specific therapies (*n* = 13) such as cognitive behavioral therapy and prolonged exposure therapy, or on training around how to work with specific age groups (*n* = 10), such as children and adolescents.

## 4. Discussion

The evaluation of this training workshop in evidence-based treatments for trauma-related mental health issues is the first to our knowledge to be systematically examined in St. Lucia or the Caribbean region more broadly. The study shows significant interest, utility, and impact of such a brief training offered to a variety of stakeholders engaged in care and advocacy of stakeholders in this nation. Indeed, our main analyses revealed a significantly improved knowledge around trauma, its prevalence, evidence-based treatments for its psychological sequalae, lower stigma towards trauma survivors, and high perceived abilities to engage in better self-care as providers for survivors of trauma, with medium to high effect sizes across these outcomes. The use of a mixed method approach that combined quantitative and qualitative data in a fairly powered sample of 41 participants supports the interpretation that such a brief training holds promise and is potentially effective for improving the knowledge and skill base of providers of trauma survivors in St. Lucia. Furthermore, the diversity and heterogeneity of the participant pool (which included a range of pertinent stakeholders, from school counselors, to nurses, to specialized psychologists, to advocates, to public health educators) increases the external validity of these findings and provides greater confidence in the ability of such a brief training to provide support to individuals involved in all areas of support for survivors of trauma.

Furthermore, given the dearth of information in the greater Caribbean region around (1) effectiveness of training programs for providers of trauma, and (2) the current rates of utilization of any evidence-based psychological therapies for trauma-related mental health issues, the creation, implementation, and concurrent evaluation of such a one-day training workshop is of paramount importance. As noted earlier, global trauma exposure rates are approximately 70% [1], with reported rates for individuals in the Caribbean being similar to or higher than these global rates [7,8]. Thus, the need for more widespread psychoeducation and skills training opportunities for providers in this region cannot be over-emphasized. In addition, similar to other low-resource settings elsewhere in the world, there is a growing need to provide mental health training to front-line providers in order to start engaging in task-shifting [17,18], which prescribes the need to train individuals who are not necessarily mental health professionals in evidence-based treatments, but rather who are embedded in other larger healthcare systems or other settings that serve as frontlines (e.g., advocates, school counselors) for trauma survivors. As a result, this workshop was designed to allow the training team to train a range of stakeholders involved in survivor care and support, as a way to engage in this process of task-shifting so that a wider range of potential providers could provide psychoeducation about post-trauma symptoms, immediate crisis relief, and referrals or information about remote resources for longer-term evidence-based psychological care for PTSD and other post-trauma reactions.

It is important to note that another key element of effective community-based and collaborative public health efforts is the involvement of the community partners as equal contributors to the knowledge exchange [29]. To this end, the training team initially proposed a trauma education and brief skills training only to the stakeholders on the island. However, over a series of conversations with survivor advocacy group leaders and governmental health educators, it became clear that a major unmet need in the domain of survivor care revolved around provision of self-care resources and strategies for these front-line providers, which is why the self-care module and techniques were incorporated into this training workshop. The qualitative data analysis supported the highly regarded utility of this added aspect by workshop attendees, and this workshop, therefore, provides a successful model for future collaborative trainer-stakeholder mental health training efforts in the region. In addition, part of the deliverables from this workshop entailed the generation of a group-think resource guide of local mental health treatment and support services provided by each of the stakeholder groups given the variety and richness of stakeholders involved in this training, which the training team collated and distributed to all the attendees and leaders of the various organizations who participated as an additional resource after the workshop.

While this workshop provided a number of positive features and strengths, it was certainly not without its limitations. The primary one was the inability to include all of the stakeholders and community providers who wished to obtain this training due to a lack of space and funding, which included some 60 providers who had to be waitlisted for this workshop. In addition to these waitlisted individuals, several other pertinent community organizations were not even approached regarding their interest in the study, despite considerable potential utility of such a training (e.g., the police force, medical first responders, the Red Cross). This indicates room for a future, more expansive workshop that can more thoroughly address this significant interest and need for this training in the community. In fact, due to this unmet need and evidence of success of the first workshop, the training team has been able to secure funding to repeat this training with a larger group, which will aim to incorporate/elaborate on some of the workshop elements highlighted by participants from this first workshop as being most helpful (e.g., more time for discussion, continued self-care instruction and practice, continued incorporation of case examples).

In addition, this training opportunity was limited to providers in St. Lucia. Given that the wider Caribbean region experiences some of the same lack of training resources (coupled with similarly high trauma exposure rates), it would have been ideal to provide this training (either in-person or remotely) to providers in other countries in the region as well, and to utilize advances in telehealth to do so [30], which was not a feature of this workshop. To this end, the training team has made technological provisions to both record the next workshop and to live stream it to providers in surrounding islands to expand the reach of this training to the broader region, and future evaluation efforts will compare the effects of such a remotely delivered training to those receiving the training in-person. Relatedly, integration of technology could also potentially be used to address a missing feature in the current study: The lack of evaluation of longer-term impacts of the training on retention of knowledge gains, actual practice effects when working with survivors of trauma, and self-care behaviors. Monitoring of long-term effects of such trainings and clinical research in general are desirable in order to yield more extensive outcome data if done in the most methodologically sound way [31].

Finally, it is notable from a scientific standards’ perspective that the measures used to evaluate effectiveness and utility of this training workshop were not validated (as is typically recommended, [32]) and were specifically created as evaluative tools for this workshop. This decision to use specifically created measures was not made lightly, given the obvious drawbacks to such an approach in terms of potential threats to psychometric rigor. However, there were several major reasons that ultimately made this approach necessary. First, there currently is a lack of validated measures that are appropriate for use in such global settings for some of the concepts examined (e.g., assessment of increases in knowledge about trauma and its correlates, or knowledge about evidence-based treatments for trauma-related mental health issues). Second, the primary aim of these pre- and post-workshop questionnaires was not conceived as a traditional research assessment, but rather as an evaluative assessment of the workshop’s effectiveness, impact, and acceptability to the population of providers examined in this global context (for whom very little information exists around current knowledge and psychology training, as noted previously). However, the handful of studies that have examined similar concepts in trauma treatment providers outside of this region (primarily in the United States) have created questionnaire batteries that are very close in content, using a similar approach to generating items that most closely mirror the constructs of interest, namely effectiveness of training efforts in improving knowledge around trauma and PTSD and expanding familiarity with evidence-based treatments for this area [33,34]. In addition, input from collaborators on the ground was sought out to ensure the final questionnaires inquired about important aspects highlighted and common to the range of stakeholders attending this training (since the participants were not uniform in their occupation or level of training in trauma), specifically knowledge about trauma and its correlates, and stigma towards trauma survivors. Third, given the less common use of research procedures in this region [11,12], we wanted to ensure the burden of pre- and post-workshop questionnaires was not too high on participants. Thus, we opted to prioritize only asking the questions most directly linked to the outcomes of greatest interest, instead of adding a number of measures that contained elements not relevant to the current study.

Future examinations of such training workshops in the region, therefore, have several distinct areas to consider to improve on the current design. For one, whenever possible, future studies in this and other global settings are strongly advised to include validated measures that are related to the concepts examined here (such as stigma towards trauma survivors), as long as the benefits of such measurement are not overly burdensome to the populations examined. This is a feature the authorship team intends to include in such future training evaluations wherever available and appropriate. A second consideration is to include a longer-term follow-up assessment (such as one occurring a week, a month, or three months after the workshop) of more distal outcomes as a result of such brief trainings. This could include maintenance of gains in knowledge around trauma-related pathology and their effective treatments, practice differences in terms of implementation of the skills taught (to others or themselves as providers), and dissemination of their own training to other providers/colleagues, among others. These longer-term assessments could be conducted over a remote platform (such as an online survey completed at the participants’ convenience) or via the telephone to reduce burden on participants. Another area for future work includes taking into account what other researchers conducting work in this region have strongly suggested: The need to integrate such trainings as what was done here into a larger healthcare system in order to promote sustainability and maximal positive effect [35]. To this end, this study closely partnered with the Ministry of Health and Wellness on the island, in order to provide this training to health educators embedded in larger healthcare systems on the island, although the dissemination of this training across these systems was not explicitly discussed, which would be fruitful to do moving forward with this and other similar collaborations.

## 5. Conclusions

This study is the first to our knowledge to systematically examine the impact of a one-day psychoeducation and training workshop for providers supporting trauma survivors in Saint Lucia. This relatively short training was of high interest, effectively improved knowledge around trauma and evidence-based treatments, and reduced stigma towards trauma survivors across a variety of stakeholders engaged in the care of trauma survivors. Given the significant dearth of evaluative or research studies of mental health resources in this country and the wider Caribbean region [12], this study significantly contributes to increasing understanding around ways to reduce the significant mental health disparities related to trauma in this particular global setting. Further, the focus of this training on including a variety of non-psychology specialists in order to engage in task-shifting has been recognized as an effective way to further close this gap between a high need for services for trauma survivors and a low prevalence of knowledgeable providers in the area [17]. This training endeavor also highlighted the importance of working closely with community partners [29], and the advantages of genuinely prioritizing the equal partnership of the researcher and community teams in order to generate the stakeholder buy-in and training content that are most beneficial in addressing the mental health inequalities in such global contexts.

## Figures and Tables

**Table 1:** Demographic characteristics of workshop attendees.

Variable	Mean	SD
Age (range 20–56 years)	38.0	9.4
Variable	*n*	Percent (%)
Gender		
Female	34	85.0
Male	5	12.5
Type of organization/role		
School-based guidance counselor/Department of Education	12	30.0
Bureau of Health Education	5	12.5
Other Government Ministry/Department	4	10.0
Registered Nurse or Affiliated Hospital provider	8	20.0
Non-governmental organization (NGO) or Advocacy organization	10	25.0
Highest degree earned		
Completed Secondary (High) School	2	5.0
A-levels or Associates Degree	3	7.5
Bachelor’s Degree	9	22.5
Master’s Degree	18	45.0
Post-Master’s Degree	4	10.0
Other	2	5.0
Years of experience working with trauma survivors		
0–2 years	8	20.0
3–5 years	2	5.0
6–10 years	8	20.0
10+ years	9	22.5

**Table 2:** Correlations between demographic characteristics and self-reported trauma knowledge, trauma stigma, and self-care prior to workshop.

Variables	1.	2.	3.	4.	5.	6.	7.	8.	9.	10.	11.	12.
1. Age	-											
2. Gender	0.377 *											
3. Highest Education	0.343 *	0.050										
4. Years of Experience	0.343	0.194	0.395 *									
5. Trauma-related burnout	0.024	−0.018	0.133	0.281								
6. Self-care support	0.069	−0.053	0.197	−0.301	−0.230							
7. Self-care value	0.148	0.044	0.233	0.190	−0.109	0.061						
8. Self-care behavior	−0.043	−0.075	−0.083	0.104	0.084	0.001	0.193					
9. Knowledge of Trauma	0.218	0.173	0.203	0.531 ***	−0.024	−0.042	0.683 ***	0.385 *				
10. Trauma Prevalence	−0.070	−0.008	0.175	0.363	0.184	−0.116	0.148	−0.077	0.234			
11. Stigma regarding Trauma	0.272	0.198	−0.049	0.190	0.210	0.171	−0.227	−0.031	−0.268	−0.298		
12. Knowledge of Trauma Treatment	0.028	0.187	−0.019	0.349	0.154	−0.098	−0.036	0.013	0.467 **	−0.138	−0.064	
M	38.000	*-*	*-*	*-*	5.71	5.13	9.46	7.15	24.00	10.67	3.85	5.13
SD	9.424	-	-	-	2.45	2.46	1.21	1.83	4.49	3.38	4.23	2.26

Note: *** *p* < 0.001, ** *p* < 0.01, * *p* < 0.05. Gender, highest education, and years of experience are categorical variables and, therefore, Mean (M) and Standard Deviation (SD) were not calculated for these variables. *Knowledge of Trauma* subscale was scored out of a maximum of 30. *Trauma Prevalence* was scored out of a maximum of 20. *Stigma regarding Trauma* was scored out of a maximum of 20. *Knowledge of Trauma Treatment* was scored out of a maximum of 10.

**Table 3:** One-way repeated measures analysis of variance (ANOVA) examining change in trauma subscale scores from pre- to post-workshop.

Sub-Scales	W	F	df1	df2	ES
Knowledge of trauma	0.83 *	5.11	1	24	0.18
Prevalence of trauma	0.70 **	14.64	1	34	0.30
Stigma towards trauma	0.85 *	6.58	1	37	0.15
Knowledge of trauma treatment	0.32 ***	74.54	1	35	0.68

Note: (ES) = Effect size. * *p* < 0.05; ** *p* < 0.01; *** *p* < 0.001.
